# *Schistosoma haematobium* Infection and CD4+ T-Cell Levels: A Cross-Sectional Study of Young South African Women

**DOI:** 10.1371/journal.pone.0119326

**Published:** 2015-03-13

**Authors:** Elisabeth Kleppa, Kari F. Klinge, Hashini Nilushika Galaphaththi-Arachchige, Sigve D. Holmen, Kristine Lillebø, Mathias Onsrud, Svein Gunnar Gundersen, Myra Taylor, Patricia Ndhlovu, Eyrun F. Kjetland

**Affiliations:** 1 Norwegian Centre for Imported and Tropical Diseases, Department of Infectious Diseases, Oslo University Hospital, Oslo, Norway; 2 Faculty of Medicine, University of Oslo, Oslo, Norway; 3 Department of Gynaecology, Oslo University Hospital, Oslo, Norway; 4 Research Unit, Sorlandet Hospital, Kristiansand, Norway; 5 Department of Global Development and Planning, University of Agder, Kristiansand, Norway; 6 Discipline of Public Health Medicine, Nelson R Mandela School of Medicine, College of Health Sciences, University of KwaZulu-Natal, Durban, South Africa; 7 Imperial College, London, United Kingdom; Johns Hopkins University, UNITED STATES

## Abstract

*Schistosoma (S*.*) haematobium *causes urogenital schistosomiasis and has been hypothesized to adversely impact HIV transmission and progression. On the other hand it has been hypothesized that HIV could influence the manifestations of schistosomiasis. In this cross-sectional study, we explored the association between urogenital *S*. *haematobium* infection and CD4 cell counts in 792 female high-school students from randomly selected schools in rural KwaZulu-Natal, South Africa. We also investigated the association between low CD4 cell counts in HIV positive women and the number of excreted schistosome eggs in urine. Sixteen percent were HIV positive and 31% had signs of urogenital schistosomiasis (as determined by genital sandy patches and / or abnormal blood vessels on ectocervix / vagina by colposcopy or presence of eggs in urine). After stratifying for HIV status, participants with and without urogenital schistosomiasis had similar CD4 cell counts. Furthermore, there was no significant difference in prevalence of urogenital schistosomiasis in HIV positive women with low and high CD4 cell counts. There was no significant difference in the number of eggs excreted in urine when comparing HIV positive and HIV negative women. Our findings indicate that urogenital schistosomiasis do not influence the number of circulating CD4 cells.

## Introduction

Helminth infections and HIV coexist in areas where the diseases’ burdens are high [1]. *Schistosoma (S*.*) haematobium* causes urogenital schistosomiasis that has been shown to be associated with HIV in cross-sectional studies [2–4]. In Sub-Saharan Africa, *S*. *haematobium* infection is correlated with HIV at a country level [5]. Schistosomiasis has been hypothesized to adversely impact every phase of HIV / AIDS, e.g. to increase the susceptibility to HIV, influence HIV viral load and further disease progression [3,6].

It has been proposed that schistosomiasis and other helminths alter the immune system by skewing it towards a Th2-type response [7]. Chronic helminth infections may therefore promote HIV-1 pathogenesis [7,8]. Few studies have investigated the effect of helminth infections on CD4 cell count in HIV negative, and little is known about possible systemic effects of schistosomiasis on the immune system [9]. Furthermore, it has been hypothesized that having a lower CD4 cell count before HIV seroconversion may accelerate the progression to AIDS [10].

**Fig 1:**
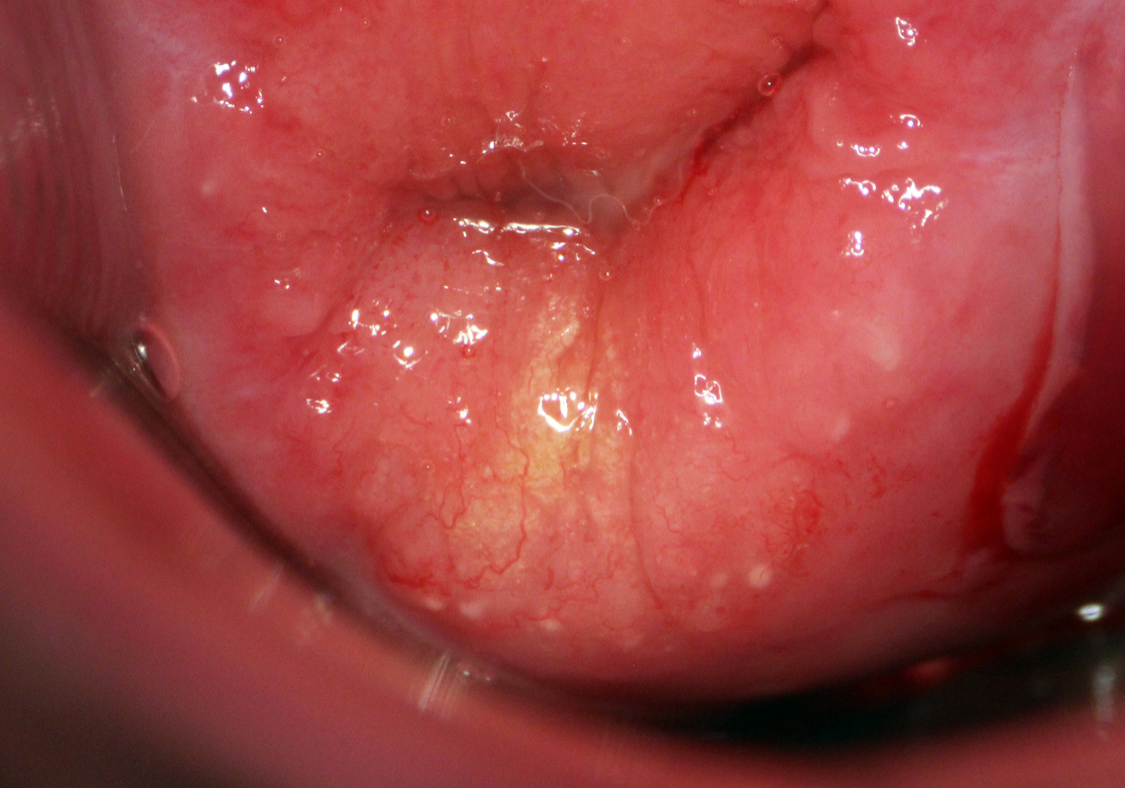
Sandy patch on the cervix. Colposcopic image of the ectocervix showing a grainy sandy patch at 6 o’clock. In addition there are many scattered grains in a sector from 4 o’clock to 7 o’clock. Abnormal blood vessels can also be seen on the mucosa, many near and between the schistosome grains.

Genital *S*. *haematobium* infection in women is characterized by so-called sandy patches that can appear grainy or homogenous and are thought to be caused by the deposition of schistosome eggs in the genital tissues (Fig. 1) [11]. Grainy sandy patches are considered pathognomonic for genital schistosomiasis [2]. Sandy patches are often associated with abnormal blood vessels and contact bleeding due to the fragile mucosa [2].

However, *Schistosoma haematobium* is usually explored in urine only [12]. It has been hypothesized that egg excretion in urine is higher in persons with a functioning immune system, but the evidence is conflicting [13–15]. In the largest study done on this topic, in Zimbabwe, Kallestrup et al. did not find decreased egg excretion in participants co-infected with HIV [13].

To our knowledge, no previous studies have investigated CD4 cell counts in women with urogenital schistosomiasis.

In this study, we explored the association between urogenital *S*. *haematobium* infection and CD4 cell counts in HIV positive and negative young women. We also investigated the association between low CD4 cell counts in HIV positive and the number of excreted schistosome eggs in urine.

## Materials and Methods

### Study design and population

This cross-sectional study took place from May to October 2013. The participants were part of a cohort of female high-school students included in a prospective study on female genital schistosomiasis in rural KwaZulu-Natal, South Africa. All women who were sexually active and above the age of 16 years were invited to participate in the study. CD4 cell counts and HIV testing were done in all study participants seen during this period. Women from 26 randomly selected high schools were included. Pregnant women were not investigated.

This rural district on the East coast is one of the poorest in South Africa, endemic for urogenital schistosomiasis and with a high prevalence of HIV [16,17].

### Clinical investigation

The gynecological examinations were performed by trained female medical doctors. The entire cervicovaginal surface was examined colposcopically using an Olympus OCS 500 Colposcope with a mounted Olympus E420 (10 Mpx) or a Leisegang colposcope with a mounted Canon EOS 650D (18 Mpx). Lesions indicating genital schistosomiasis were recorded as sandy patches with or without the presence of abnormal blood vessels [2].

### Laboratory analyses

A urine sample was collected between 10 a.m. and 2 p.m. to ensure optimal egg yield [18]. Merthiolate-formalin solution (2%) was added to 10 mL of urine. Urine samples were centrifuged and the whole pellet was deposited on one or more glass slides. Schistosome eggs in urine were counted by microscopy by trained research assistants. The mean egg intensity (eggs / mL) was calculated by dividing the total number of eggs in a urine sample by the volume. A positive diagnosis was recorded if at least one egg was seen.

HIV testing was done using Bioline Rapid Test HIV (NJ, US) and confirmatory Sensa Tri-Line HIV Test Kit (Pantech, Durban, South Africa).

Blood was collected in EDTA tubes for CD4 analyses and serum samples for frozen storage (Vacutainer tubes, Becton, Dickinson and Company (BD), Franklin Lakes, NJ, US). The EDTA tubes were transported to the NHLS laboratory, Stanger Hospital, accredited by the SA National Accreditation System (SANAS)—an independent legislated body for compliance with international standards (ISO 15189:2007). CD4 cell count analyses were performed within 24 hours by a Beckman Coulter flow cytometer (Cytomics FC 500, Brea, California, US).

### Ethical considerations

Research ethical committees in South Africa and Norway approved the study (Biomedical Research Ethics Committee (BREC), University of KwaZulu-Natal (Ref BF029/07), KwaZulu-Natal Department of Health, (Reference HRKM010–08) and the Regional Committee for Medical and Health Research Ethics (REC), South Eastern Norway (Ref 469–07066a1.2007.535)). Furthermore, the Departments of Health and Education in Ugu district, KwaZulu-Natal gave permissions for this study. All participants signed individual, written informed consent forms. The ethical committees, BREC (annual renewal) and REC, were aware that minors were participating in the study and specifically approved the consent procedure (independent minor consent, no parental consent). STI treatment was offered in accordance with the South African syndromic treatment protocol [19]. Anti-schistosomal treatment was offered to all as part of a mass drug administration campaign. All participants were tested for HIV, but disclosure of the result was voluntary. HIV testing and follow-up was done in accordance with South African guidelines with pre- and post-test counseling. If the CD4 cell count was below 350 x 10^6^ cells / L, the patient was contacted by an HIV counselor, and taken to her local clinic for follow-up according to official South African guidelines. HIV negative women with a CD4 cell count below 350 x 10^6^ cells / L were asked to come for repeat tests for both HIV and CD4 cell count. HIV positive patients were referred to local clinics for follow-up and treatment. South Africa provides universal access to antiretroviral treatment (ARV).

### Statistical analyses

Statistical analyses were performed using IBM SPSS Statistics version 20 (Armonk, NY, USA).

Sample size calculation showed that in a population with 20% urogenital schistosomiasis (assumed prevalence), we needed to recruit 600 participants in order to detect a difference in mean CD4 cell count of 80 x 10^6^ cells / L with a power of 80% [20]. A 95% interval of confidence was used throughout.

The CD4 cell counts followed a close to normal distribution. Stratifying for HIV status, we used the Student’s t-test to compare mean CD4 cell counts for women with and without sandy patches, abnormal blood vessels and schistosome eggs in urine.

In HIV positive women, we defined three groups by CD4 cell count (below 350, 350–500 and above 500 x 10^6^ cells / L). We used analysis of variance (ANOVA) when comparing these groups’ prevalences of sandy patches, abnormal blood vessels and schistosome eggs in urine. Egg counts were log transformed in order to obtain normal distribution. Linear regression was used when investigating the association between mean egg intensity in urine and CD4 cell count.

## Results

CD4 cell counts were performed in 792 students from 26 rural high schools. The mean age was 18.9 years (standard deviation (SD) 2.2). We obtained HIV tests from 765, of which 123 were HIV positive (16.1%). The mean age for HIV positive women was 20.1 (SD 2.4) years as opposed to 18.7 (SD 2.1) years for the HIV negative. Only 30.1% of the HIV positive (37/123) reported that they knew they were HIV positive, and 54.1% of these (20/37) reported to be on ARVs. As expected, the HIV negative group had a significantly higher mean CD4 cell count than the HIV positive group (931 [SD 289, range 236–2549] vs. 512 [SD 243, range 60–1359] x 10^6^ cells / L, p < 0.001).

The clinicians reported genital sandy patches in 78 women (12.2%) and abnormal blood vessels in 294 women (45.8%). We found schistosome eggs in urine samples from 154 participants (20.0%). In total, 208 of 665 women (31.3%) had either sandy patches or *S*. *haematobium* eggs in urine.

### Genital lesions, urinary schistosomiasis and CD4 cell count

**Fig 2:**
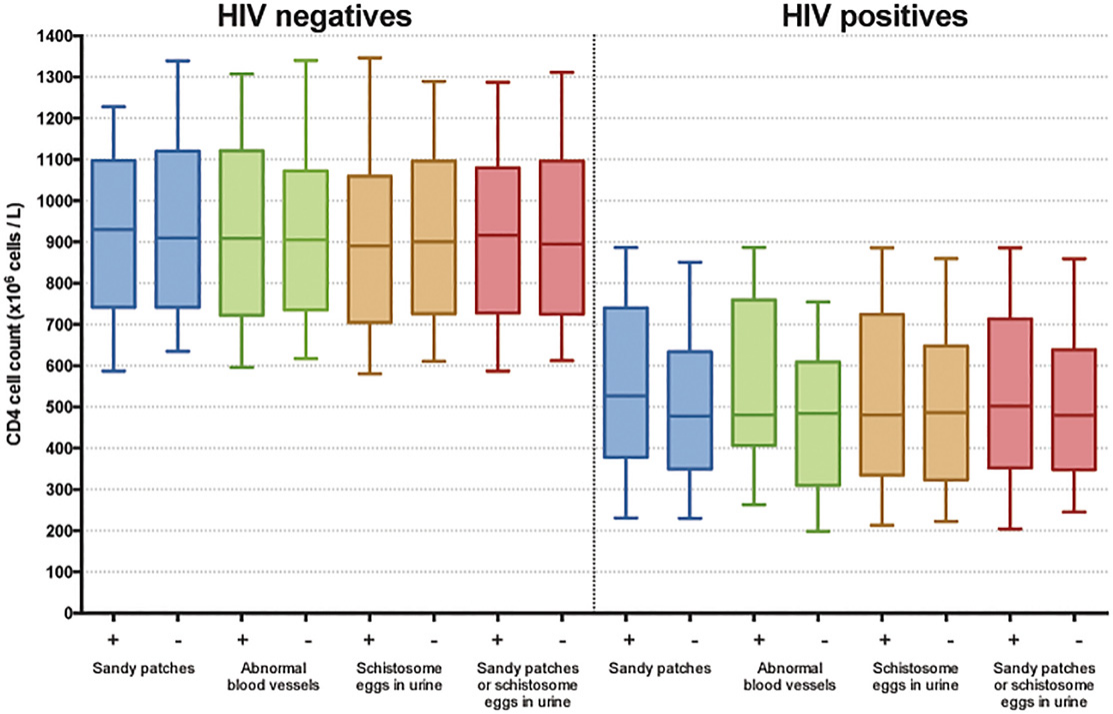
Genital lesions, urinary schistosomiasis and CD4. Boxplots showing CD4 cell counts in HIV negative and positive women with different findings of urogenital schistosomiasis. The whiskers represent the 10th and 90th percentiles. The mean CD4 cell counts showed no significant differences between any of the groups.

There was no significant difference in CD4 cell counts between the participants with and without sandy patches, abnormal blood vessels or schistosome eggs in urine after stratifying for HIV status (Fig. 2). Also after combining the findings of genital sandy patches and / or eggs in urine, we found no difference in mean CD4 cell count (Fig. 2).

### Schistosomiasis prevalence and low CD4 cell count

**Fig 3:**
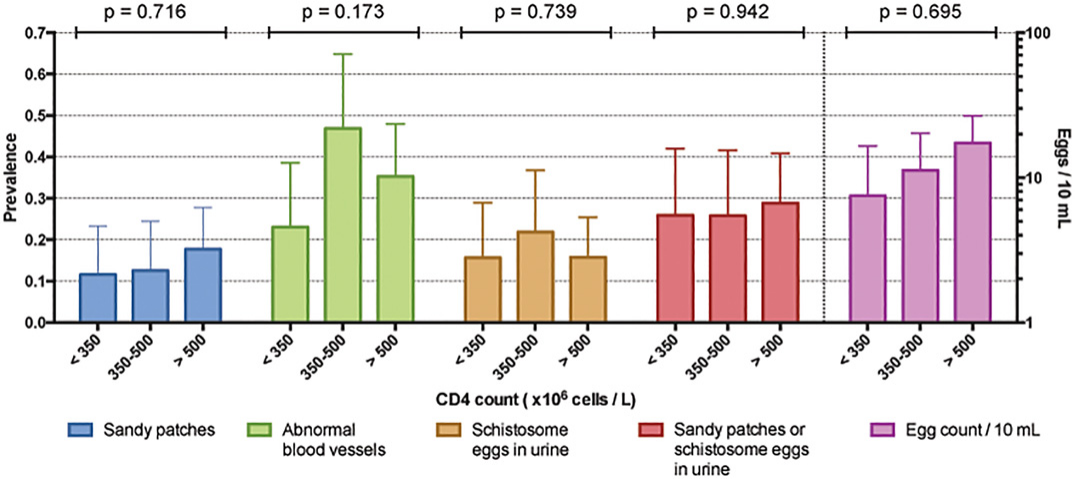
Urogenital schistosomiasis and CD4 in HIV positive. HIV positive women were grouped by CD4 cell count (< 350, 350–500 and > 500 x 10^6^ cells / L). Mean prevalence of findings related to urogenital schistosomiasis was compared across the groups using analysis of variance (ANOVA). The resulting p-value is indicated above each group. Error bars represent 95% confidence intervals. No significant difference was found for any variable across the CD4 groups.

The prevalences of sandy patches, abnormal blood vessels and schistosome eggs in urine in HIV positive women were similar across three groups defined by CD4 cell count (below 350, 350–500 and above 500 x 10^6^ cells / L). The results are presented in Fig. 3.

### *Schistosoma* egg excretion (intensity) and CD4 cell count

**Fig 4:**
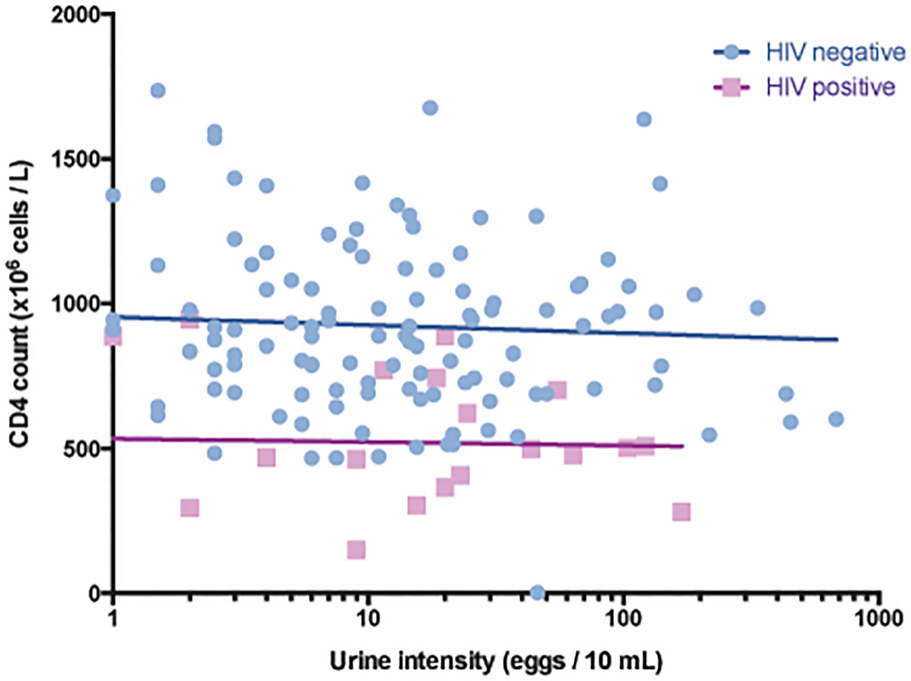
Egg excretion and CD4. Scatterplot showing CD4 cell counts and urine schistosome egg intensity in HIV negative and positive women. Only women excreting schistosome eggs (n = 156) were included. Regression lines were fitted for each of the groups. No linear associations were found.

In women with schistosome eggs in urine, there was no significant difference in mean egg excretion between the HIV positive and HIV negative (12.3 and 10.9 eggs / 10 mL, respectively, p = 0.760). Furthermore, we did not find any significant difference in mean egg excretion in HIV positive across the three groups defined by CD4 cell count (Fig. 3). In a regression analysis including participants excreting eggs, there was no significant linear association between egg excretion and CD4 cell count in HIV negative or positive women (r^2^ = 0.002 and 0.001, p = 0.665 and p = 0.869, respectively, Fig. 4).

## Discussion

In this study, we did not find a significant difference in mean CD4 cell count of women with and without urogenital schistosomiasis (genital lesions and / or urinary schistosomiasis), neither in the HIV negative nor positive group. Further, we did not find a significant difference in urine *S*. *haematobium* egg intensities when comparing HIV positive women with low and high CD4 cell counts.

CD4 cell counts are rarely explored in HIV negative individuals, and this was the first study to explore the effects of *S*. *haematobium* on CD4 cell count comprehensively [21–24]. It may be surprising that there was no significant difference in CD4 cell count between women with and without urogenital schistosomiasis, as the infection causes systemic pathological effects [9]. However, studies on the effects of helminths on CD4 levels have shown various results [21,23–25]. An Ethiopian study did not find any association between intestinal parasitic infections and CD4 cell count [26]. Likewise, a Ugandan study of HIV infected persons did not find lower CD4 cell counts in those co-infected with *S*. *mansoni* [23]. Furthermore, anthelminthic therapy did not show any significant impact on the CD4 cell count [23]. However, Kallestrup et al. included both HIV positive and negative persons to a randomized controlled trial, and found that praziquantel treatment was associated with a slight increase in CD4 cell count in HIV positive women [21]. A Cochrane analysis on the treatment of HIV positive with helminth co-infection concluded that anti-helminthic treatment may slow the decline in CD4 cell count [27]. However, a review including observational data did not find that treating helminth infections improved the CD4 cell count [28].

Our findings indicate that the mean CD4 cell count in women was not influenced by schistosomiasis or that the level of egg excretion was influenced by CD4 cell count. There were few adult survivors of mother-to-child transmission of HIV at the time of the study, and relatively recent sexual transmission was most likely in this young population. This may be the reason we had few subjects with very low CD4 cell counts, and none below 50 x 10^6^ cells / L. The findings might therefore represent a Type 2 error due to a limited sample size or sub-optimal sensitivity and specificity of the investigations. Several clinicians investigated women, and we cannot rule out the possibility of inter-observer variability in the colposcopic examinations. A computer image analysis of lesions may improve the FGS diagnosis (Holmen et al., submitted).

Stool samples were not investigated in our study and hence we were not able to assess the possible effects of soil-transmitted helminths on the CD4 cell count. Sexually transmitted infections, such as syphilis, may also influence the CD4 cell count [29]. *S*. *mansoni* was not likely to be a confounder, since only very scanty pockets of this species are found in this area of South Africa. We only collected one urine sample and the intensity of infection (mean egg count) and the prevalence of *S*. *haematobium* might be higher [30]. A more sensitive test, such as circulating anodic antigen (CAA), would have given an indication of the number of women with live schistosomiasis worms [31]. Helminth infections are often present in the host for years and regulate the immune system, but effects may be subtle and vary between helminth species [32,33]. Increased numbers of HIV target cells have been found to be associated with genital schistosomiasis in cervical samples, and these local immune responses may be of greater importance to the susceptibility to HIV than the systemic effects [34,35]. It is also possible that *S*. *haematobium* infection affects the proportions of CD4 T-cell sub-populations [36] without changing the CD4 cell count significantly.

In one study of HIV positive individuals, several parasitic infections occurred more frequently in those with low CD4 cell counts, as is the case with opportunistic infections [37]. But it has also been hypothesized that chronic parasitic infections themselves shift the T-cell populations and decrease the CD4+ cell count [24,33]. Immune deficiency may influence egg excretion, but the evidence is conflicting [13–15]. We did not find any indication of lower egg excretion in the urine of HIV positive women, even with low CD4 cell counts. Furthermore, we found similar numbers of genital lesions in women with low CD4 cell counts. The described association between HIV and urogenital schistosomiasis [3,4] was therefore not likely to be influenced by altered egg excretion in HIV positive. Further studies are warranted to rule out whether severe immunodeficiency may influence egg excretion in urine and / or the appearance of genital lesions.

## Supporting Information

S1 File Dataset with relevant variables. (SAV)
